# The perceptions of Dutch general practitioners on the implementation of a proactive integrated care approach for patients with complex needs: a pilot survey

**DOI:** 10.1186/s12875-026-03176-x

**Published:** 2026-01-16

**Authors:** Kimberley A. Leming, Laurens C. van Gestel, Marc A. Bruijnzeels, Rimke C. Vos, Marieke A. Adriaanse

**Affiliations:** 1 Health Campus The Hague/Department of Public Health and Primary Care, Leiden University Medical Center, Turfmarkt, 99, 3rd floor, The Hague, 2511 DP the Netherlands; 2 Health, Medical and Neuropsychology Unit, Leiden University, Leiden, the Netherlands

**Keywords:** Patients with complex needs, Primary care, Exploratory study, Integrated and proactive care

## Abstract

**Background:**

For a subgroup of patients with complex health needs on multiple domains (somatic, mental and/or social) often accompanied with disproportional healthcare use, a new proactive and integrated care (PIC) approach is being developed in the primary care setting to stimulate health improvement among these patients. To ensure sustainable implementation of the PIC approach it is important to study factors influencing the initial uptake of the intervention prior to actual implementation. This Pilot survey explores whether general practitioners (GPs) recognize patients with complex needs, understand their needs, and how they perceive the acceptability, appropriateness, and feasibility of the PIC approach.

**Methods:**

For this pilot survey, a convenience sample of GPs was recruited in the regions of Utrecht and The Hague in the Netherlands. They completed a questionnaire assessing their recognition and awareness of these patients’ needs, and their views on the acceptability, appropriateness, and feasibility of the PIC approach.

**Results:**

In total 22 GP filled in the survey. Most GPs (90.90%) recognize patients with complex needs as a patient group, yet only 42.90% report awareness of their needs. Furthermore, 80.90% agree that care change is needed. While only 4.50% of GPs find the PIC approach to be unacceptable, a larger portion express doubts about its appropriateness (22.70%) and feasibility (22.70%).

**Conclusions:**

As an exploratory pilot survey, these findings offer initial indicators that while GPs acknowledge patients with complex needs and support care change, further research is needed to confirm the appropriateness and feasibility of the PIC approach.

**Supplementary Information:**

The online version contains supplementary material available at 10.1186/s12875-026-03176-x.

## Background

Globally, there has been an increase in the number of patients with complex (health)care needs [1]. Within this group of patients there is a subgroup clustered in geographical areas who experience complex problems across multiple life domains (somatic, mental and social), make frequent use of the healthcare system, and generate high healthcare costs [2, 3]. Gawanda introduced the term ‘Hotspotters’ for these patients, referring to the geographical areas where they tend to cluster [3]. Research showed that this group of patients with this level of complex needs is usually small, but nevertheless responsible for a large portion of overall healthcare costs [3, 4]. To illustrate, Wammes et al. [4] did a systematic review on a group they called “high-cost” patients. This group was labelled as the sickest and most complex population, experiencing high care utilization primarily due to their high levels of chronic and mental illness. They found that the top 10%, 5%, and 1% of these patients accounted for respectively 68%, 55% and 24% of costs within a given year. Clearly, the complexity of the (health)care problems these patients experience does not only negatively influence the patients themselves, but also puts pressure on the healthcare system. Ironically, the current healthcare system also adds to the growing complexity of these patients by providing care that is fragmented and reactive, or otherwise known as care that results in unmet needs and more hospital admissions [5].

To better facilitate Hotspotters to improve their health and to decrease unnecessary pressure on the healthcare system, it might be helpful to adjust the current care approach to better align with the patients’ needs [4]. Previous studies aiming to improve health among patients with a similar level of complexity as Hotspotters, identify several elements that should be considered when trying to better align with the patients’ needs. That is, facilitating patients with complex needs in a more fitting manner is generally considered to entail care that is person-centered, that addresses health problems proactively rather than reactively, and that stimulates the communication between professionals from various care domains [3, 6–10]. Furthermore, it is found to be of importance that this change starts in the primary care setting, as patients heavily rely on the general practitioners (GPs), the gatekeepers of healthcare [11–14].

Even though an integrated approach dealing with both medical and welfare aspects is often suggested for complex patients. Studies show promising but inconclusive results such as improved health, better long-term self-management of disease, fewer treatment barriers and a better experience of care [15–18]. Also, feasibility of implementing an integrated approach in the Netherlands is inconclusive. Insight are based on a pilot study which was conducted in 2015 in Zoetermeer, a small city in the west of the Netherlands. This study provided patients with complex needs with alternative care based on the Positive Health Methodology [19]. This methodology was deemed particularly fitting as it aims to gain a comprehensive understanding of patients’ health by exploring various life domains, leading to a broader understanding and dialogue about health. It also can provide a more person-centered and proactive care approach, Building on the insights gained from this pilot, this alternative care approach has been expanded by incorporating multidisciplinary meetings where not only the patient is present, but also professionals from different care domains. This new addition aims to stimulate the communication between these professionals and to integrate the patient in the decision-making process pertaining to their healthcare plan. This expanded Proactive Integrated Care (PIC) approach will systematically be introduced to various general practices located in urban areas in the Netherlands as part of a new Hotspotters trial [2].

However, before introducing the PIC approach to various general practices, it is important to explore factors that might influence implementation in practice. Unfortunately, the integration of evidence-based interventions into routine daily practice does not always succeed, as research results of health services have consistently shown a gap between the evidence presented in the literature and the practical implementation [20, 21].

Next to well-known factors as organization, funding, ambiguity within integrated primary and specialist care teams about roles and responsibilities, and patients and professionals motives and behavior, increasing workload due to increased demand and shortage of staff, influence the implementation of integrated care initiatives in general practice heavily. The initial uptake of new interventions in general practices is a major concern. To address this initial uptake issue, specific implementation factors should be considered in the early phases of the intervention [22–24].

The three core implementation factors that have been found to influence the initial uptake of interventions prior to full-scale adoption in practice are acceptability, appropriateness and feasibility [24–26]. By exploring these factors among the gatekeepers of healthcare (GPs) in the early stages of setting up the intervention, we can identify whether the approach needs to be adjusted to achieve successful adoption. For example, the heavy workload and pressure on GPs in their routine daily practice might negatively affect how acceptable, appropriateness, and feasible they find the PIC approach [27, 28], which would mean that changes need to be made to the trial in order to avoid testing an intervention that will not be implemented in practice.

In light of the above, the present study aims to explore the perceptions of the GPs pertaining to the acceptability, appropriateness and feasibility of the PIC approach. In addition, and before exploring these factors, we also aim to verify whether these gatekeepers indeed recognize our target group, the so called Hotspotters, as well as the need for care change. That is, for change to happen and implementation to be successful, it is important that the professionals who are part of the implementation process recognize the value of the change [29].

## Methods

### Participants and design

We conducted a pilot survey using a convenience sample of 22 GPs. None of the GPs were in any way involved in the upcoming Hotspotters trial in an effort to guard against possible selection bias. The GP practices from our sample were located in the Dutch regions around Utrecht and The Hague. The Participants residing in the region of Utrecht were approached through a mailing list, consisting of approximately 500 actively practicing GPs (in the year 2023). This mailing list was accessible through the GP care group in Utrecht,

The GPs received an e-mail asking whether they would be willing to participate in a survey study on their perception of the PIC approach. If participants were open to participate, they could use a anonymous Qualtrics link provided in the e-mail that directed them to the questionnaire. The participants that were approached through this mailing list received one reminder. Participants residing in the region of The Hague were approached in a similar manner, with the main difference being that they were introduced to this study through a newsletter that was send out by an extra mural network from the University Medical Center in Leiden. In this newsletter they received a summary of the study and were asked whether they were willing to participate in a survey on their perception of the PIC approach in terms of acceptability, feasibility, and appropriateness.

Furthermore, a sensitivity analysis was preformed to determine the minimum detectible correlation given our sample size. The analysis indicated that, with a sample size of 22 GPs, 80% power, and a significance criterion of α = 0.05, we would be able to reliably detect a correlation of 0.567 or larger.

### Procedure

In the questionnaire we first collected demographic information. Then, we assessed our main items regarding recognizing the Hotspotters patient group, the need for care change for this group, and the implementation outcomes, followed by organizational capabilities within the general practice to implement the PIC approach Participation in the survey study lasted approximately 15 min. The Qualtrics questionnaire can be found in Additional file 1.

### Measures and materials

Outcomes were assessed on dichotomous scales (yes or no) or using a 5-point Likert scale ranging from 1 “Completely disagree“ to 5 “Completely agree“.

### Demographics

All participants were asked about their age, gender (male, female, non-binary or would rather not say), and the location (urban or rural area) of their general practice.

### Recognition of hotspotters

GPs answered the above mentioned items based on the following definition of Hotspotters that was presented to them: “Hotspotters are patients that have a complex care need and frequently use acute care due to the presence of chronic somatic problems, social problems, and/or mental problems” (see additional file 1).

Recognition of Hotspotters according to the provided description was assessed using 1 self-designed item (“I recognise the patient group in the general practice I work at”) that could be answered with “yes” or “no”. Recognition of the needs of the patient group was also assessed with 1 item (“I am aware of the needs of this patient group”) using a 5-point Likert scale.

### The need for care change and the recognition factors that influence hotspotter care

Recognition of the facilitating factors (“I am aware of the factors that could positively influence the care of these patients “), and recognition of problems pertaining to the care the Hotspotters receive (“I am aware of the problems that are part of the care approach of these patients), and the need for care change (“I think that the care approach that is used for these patients should change”) were each measured with 1 item using a 5-point Likert scale. These items were constructed for the purpose of the present study and inspired by Kegler et al. [30], who applied Damschroder et al.’s [31] Consolidated Framework for Implementation Research (CFIR) to explore themes such as organizational capacity, collaboration, and awareness of one’s target population. Although these items are simplified and tailored to the primary care setting, they reflect key CFIR domains that are relevant to understanding inner setting of care organization.

### Implementation outcomes

Given the early-stage implementation nature of the study, the implementation outcomes acceptability, appropriateness, and feasibility were measured. These outcomes were assessed with the validated Acceptability of Intervention Measure (AIM), Intervention Appropriateness Measure (IAM), and Feasibility of Intervention Measure (FIM), respectively [26]. In this study, we define acceptability as the extent to which the PIC approach is perceived as satisfactory or agreeable by the GPs; appropriateness as the perceived fit or relevance of the approach to their clinical context; and feasibility as the extent to which the intervention is seen as practical to implement within constraints. The questionnaire consisted of 12 items in total: 4 items for acceptability (e.g., “This approach seems possible”), 4 items for appropriateness (e.g., “This approach seems fitting”), and 4 items for feasibility (e.g., “This approach meets my approval”). All items were measured on a 5-point Likert scale. Previous research has established validity of these questionnaires [26]. All items were translated and back translated to Dutch from the original English version [26]. The reliability of each subscale was good (AIM Cronbach’s α = 0.94, IAM Cronbach’s α = 0.90, and FIM Cronbach’s α = 0.88). In line with previous research, we calculated the mean scores for the respective items of the subscales (cf. McGrady et al. [32]; Wong et al. [33]; Wilson et al. [34]).

Additionally, three open ended questions were added to measure why participants did or did not perceive the PIC approach as acceptable, appropriate, and/or feasible (e.g., “Can you describe why you do or do not consider this care approach to be acceptable?”).

### Organisational capabilities of the general practice

Organizational capabilities of the general practice were measured using 3 items: “A specialized mental health nurse works at the general practice I work at”, “In my general practice, we work with a professional from the social domain”, and “It is possible to plan multidisciplinary meetings in my general practice”. A focus on these aspects of care organization was chosen with the PIC approach in mind. All items were answered dichotomously (yes/no).

### Data analysis

We used the Statistical Package for the Social Sciences (SPSS) version 29.0 to generate all quantitative analyses. To explore the main and secondary aim of this study, we mainly report descriptives. Means (*M*) and standard deviations (*SD*) were reported for the average score of the AIM, IAM, and FIM scales. Median (*Mdn*) and range were reported for all single item variables. In addition, Pearson correlations (for intercorrelations between continuous variables age, AIM, IAM and FIM), and Spearman correlations (all other correlations with ordinal, single item variables) were used. For all correlations, bootstrapping with 1000 samples was applied before calculating 95% Confidence Intervals (CIs). No outliers (defined as > 3 SD) were detected. We analyzed the qualitative data thematically.

## Results

### Demographics

A total of 22 actively practicing GPs were included consisting of 12 women (54.50%), 9 men (40.90%), and 1 non-binary person (4.50%). The average age was 53.64 years *(SD =* 10.40). The majority worked at a general practice in an urban area (20 participants; 90.90%), while only 2 participants (9.10%) reported working in a rural area.

### Recognition of hotspotters and their needs

**Fig. 1:**
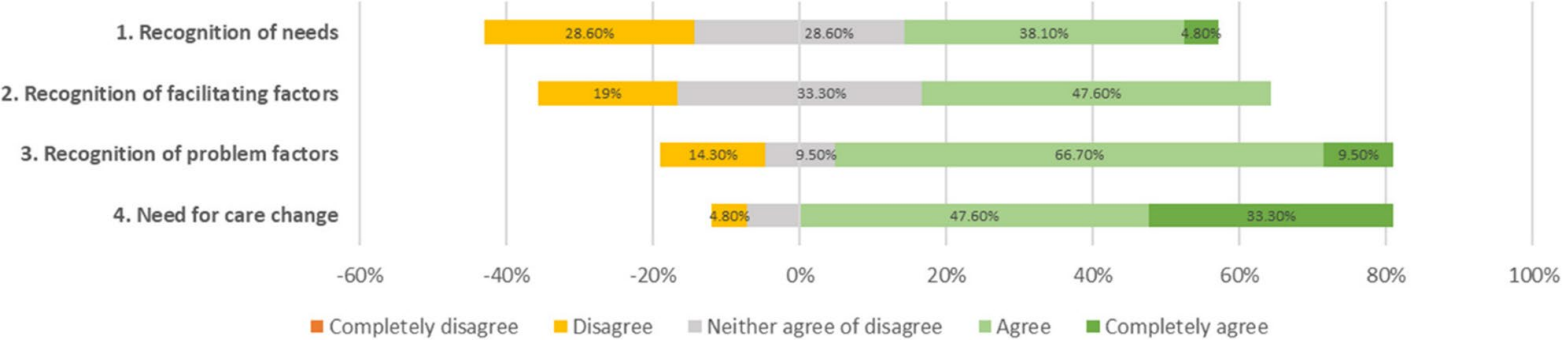
Chart with likert scale percentages of recognition of needs, need for change, recognition of facilitating factors, and recognition of problem factors

All but 2 GPs (90.90%, *n* = 20) reported to recognize Hotspotters in their general practice. Additionally, scores pertaining to the awareness of Hotspotters’ needs were around the midpoint of the scale (*Mdn =* 3.00, range = 2–5). Slightly more than a quarter of the GPs (28.60%) reported not to be aware of Hotspotters’ needs (i.e., scoring below 3; see Fig. 1 for a visual representation of the distribution of scores).

### The need for care change and the recognition factors that influence hotspotter care

Scores for awareness of the factors that positively influence the care of Hotspotters tended to be around the midpoint of the scale (*Mdn =* 3.00, range = 2–4). A minority (19.00%) indicated not be aware of the facilitators of care (i.e., scoring below 3).

Besides, GPs indicated to be generally aware of the problems in the current care of Hotspotters (*Mdn =* 4.00, *range = 2–5*). Only a small portion of the GPs (14.3%) reported not to be aware of these problem factors (i.e., scoring below 3; See Fig. 1).

Lastly, The scores for the need for care change were above the midpoint (*Mdn =* 4.00, *range* = 2–5), with just 4.80% of GPs disagreeing that the current care for Hotspotters should change (i.e., scoring below 3).

### Acceptability, appropriateness, and feasibility (AIM, IAM, and FIM)

**Fig. 2:**
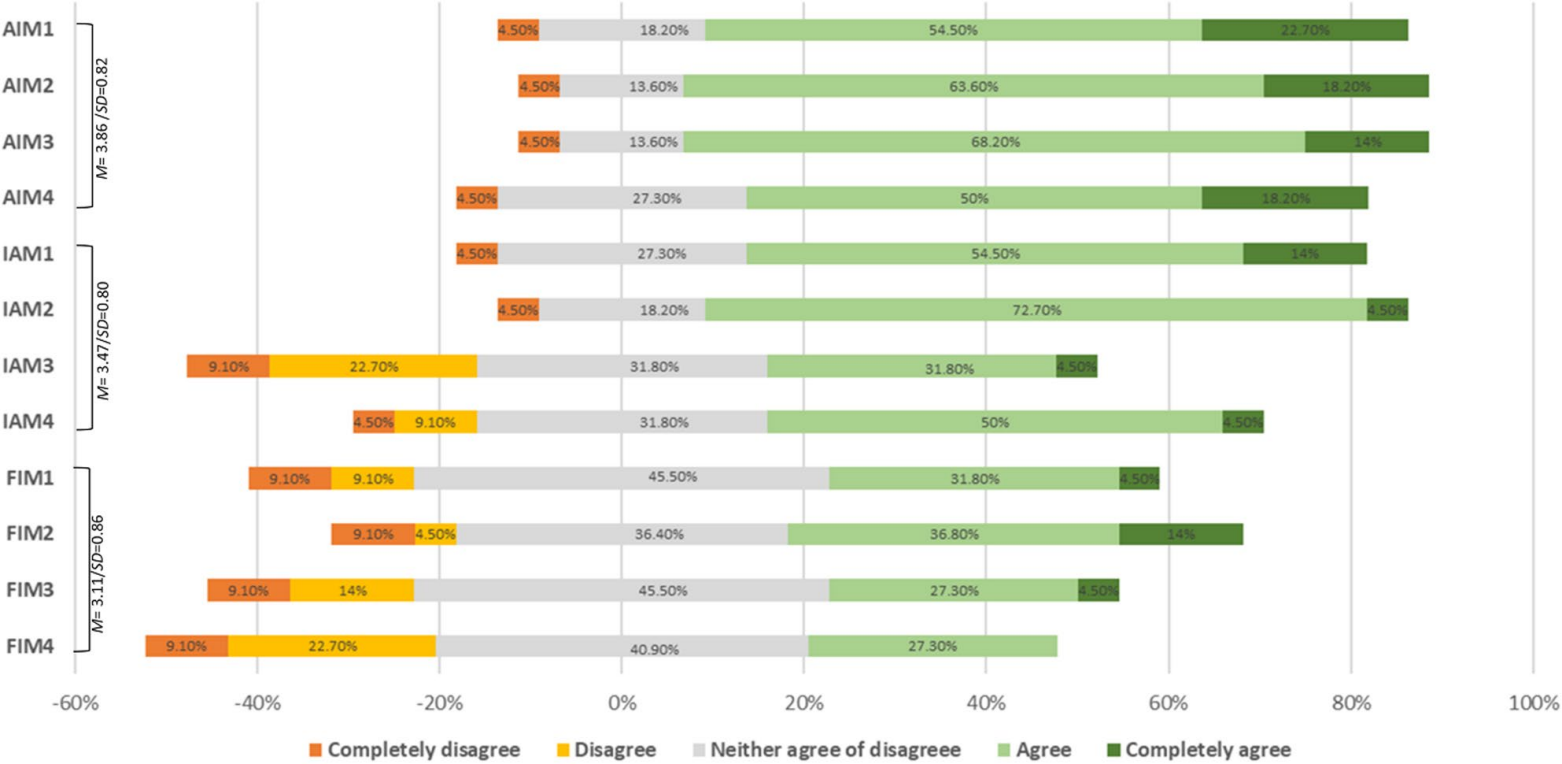
Chart containing the mean percentages on each AIM, IAM, FIM item, with the addition of SD and mean scores of each subscale

On average, GPs tended to agree that the PIC approach was acceptable (*M* = 3.86, *SD* = 0.82), with a large majority of GPs (68.20%) agreeing that this approach was acceptable (i.e., scoring 4 or above) and only 4.50% disagreeing (i.e., a score below 3). The scores for appropriateness (*M* = 3.47, *SD* = 0.80) were also well above the midpoint of the scale. However, still 22.7% had an average score that was below 3, the neutral point of the scale. The average score for feasibility was also above, but closer to the midpoint of the scale (*M* = 3.11, *SD* = 0.86), with 22.70% of participants scoring below 3 (See Fig. 2 for a visual representation of the AIM, IAM and FIM scores). Interestingly, we observe a clear shift for agree/strongly agree of about 70% of the acceptability items, to a mixture (30–70%) on the appropriateness items towards about 30% of the feasibility items.

### GPs’ perceptions of acceptability, appropriateness, and feasibility

The answers to the open-ended questions for acceptability, appropriateness, and feasibility paint a similar picture: In line with the scores on the AIM, IAM, and FIM, GPs were in general relatively optimistic. They expressed that the core elements of the intervention, such as the multidisciplinary aspect and positive health approach, could be helpful to patients and GPs in terms of time, care quality and health:

“I think that the package offered can be very pleasant for a patient and can clarify certain problem areas” (GP11).

“The approach seems very acceptable to me because care is optimized by the multidisciplinary approach. In addition, the positive health spider web ensures more initiative and direction by the patient, so that the help provided can be better matched.”’(GP2).

“…… A systemic approach and collaboration can be very satisfying for healthcare providers” (GP16).

“Investment that pays for itself in quality and time” (GP14).

Yet, (other) GPs also expressed concerns about the implementation in terms of the perceived financial burden, time investment, necessary care staff requirements, and the implementation of the approach in practice:

“The time and financial aspect makes this impossible. Currently the GPs and the mental health professionals are already overworked. Adding something that cost a lot of time makes this not feasible.” (GP20).

“……Furthermore, many projects have already been set up that have taken a lot of time, but have later fizzled out not resulting in anything” (GP18).

Finally, the above mentioned examples of GP reports also (indirectly) reflect that they are aware of certain problem factors of the current care that could be addressed with this alternative care approach. Some answers even indicated a level of awareness pertaining to the need for care change. These reports are consistent with our quantitative findings on these variables.

### Organizational capabilities of the general practice

A large majority of participating GPs (90.90%, *n* = 20) indicated that it would be possible for them to plan a multidisciplinary meeting in their general practice. Furthermore, all participants (100%, *n* = 22) reported that a specialized mental health nurse works at their general practice, and most participants (81.80%, *n* = 18) reported that there is already a collaboration set up between the general practice and the social domain.

### Intercorrelations between demographics, recognition factors and implementation outcomes

In order to further explore associations of the variables under study with the implementation outcomes, we conducted Pearson and Spearman correlations. These are reported in Table 1.

**Table 1:** Descriptives, Pearson (indicated with italics), and Spearman correlation coefficients for age, AIM score, IAM score, FIM score, gender, recognition of needs, recognition of facilitating factors, recognition of care problems, and need for care change

**Variables**	**M(SD)/%**	**1**	**2**	**3**	**4**	**5**	**6**	**7**	**8**	**9**
Age	53.64 (10.40)									
Gender	54.50% female	.191								
Recognition of needs	-	.117	-.514*							
Recognition of facilitating care factors	-	-.295	-.444	.668***						
Recognition of problem factors	-	.047	-.449*	.478*	.451*					
Need for care change	-	.066	-.213	.101	.170	.246				
AIM	3.86 (.82)	*.027*	-.156	.255	.533*	.094	.424			
IAM	3.47 (.80)	*.071*	-.275	.356	.567**	.245	.499*	.888***		
FIM	3.11 (.86)	*.314*	-.032	.314	.464*	.150	.243	.715***	.836***	

*Correlation is significant at the 0.05 level (2-tailed)

**Correlation is significant at the 0.01 level (2-tailed)

***Correlation is significant at the 0.001 level (2-tailed)

The three implementation outcomes correlated strongly with each other *(r(*20*) =* 0.72–0.89). Age and gender did not correlate significantly with implementation outcomes. Interestingly, need for change correlated moderately with all three implementation outcomes; acceptability, *r*_*s*_(19) = 0.53, 95% CI [0.17, 0.80], *p* =.013, appropriateness *r*_*s*_(19) = 0.57, 95% CI [0.15, 0.85], *p* =.007, and feasibility *r*_*s*_(19) = 0.46, 95% CI [0.12, 0.75], *p =.*034, indicating the importance of experiencing a need for change. Lastly, recognition of problem factors also correlated moderately with appropriateness *r*_*s*_(19) = 0.50, 95% CI [0.14, 0.76], *p* =.021.

## Discussion

### Summary

The aim of this pilot survey was to explore the extent to which GPs report recognition of Hotspotters as a patient group with specific needs and the need for care change as a necessary first step towards adopting the PIC approach. In addition, we aimed to explore GPs’ perceptions of acceptability, appropriateness, and feasibility of this approach. Our findings provided first insights that most GPs, in the current sample, recognize patient with complex needs. However, the GPs were divided on whether they recognize the needs of this patient group, with 27.30% of GPs reporting not to recognize their needs, and an equal part of this sample being unsure (27.30%). We also found that a majority of GPs expressed that the care for patients with complex needs should change, indicating that the current care does not meet their needs. Regarding the PIC approach, our findings showed that almost all GPs considered the PIC approach to be acceptable. GPs on average also agreed that the approach is appropriate, but still a substantial minority of GPs (varying from 4.5% to 31.8%) indicated that they did not agree that the approach was appropriate. Only a minority of GPs agreed that the PIC approach was feasible. Although most GPs reported that they had the organizational capability to implement the core elements of the PIC approach, qualitative data illustrated that GPs were especially worried about the required time, staff, and financial investment. Finally, it was found that agreement with the ‘need for care change’ was positively correlates with these implementation factors, indicating that it seems important to first addressing the urgency of care changes before suggesting alternative approaches.

### Comparison with existing literature

The present study is of an exploratory nature aimed to get better insight regarding the GPs awareness of Hotspotters, the need for care change to help this group, and their opinions on the implementability of an alternative care approach for Hotspotters. This study therefore offers future directions on these topics.

Previous research indicated that a more proactive person-centered approach with a multidisciplinary element might be more fitting for patients with complex needs [3, 6–10]. When examining how the GPs in our sample viewed the implementabillity of the PIC approach, we found that while some GPs were optimistic, a substantial minority (varying from 4.5% to 31.8%) disagreed with the implementabillity, and especially the feasibility and appropriateness of this approach. Several GPs expressed that the approach could only be implemented if certain aspects, in particular the perceived time investment, financial burden, and necessary care staff requirements were revised. This indicates that it seems that we need to carefully assess whether such elements of the approach need to be reassessed or downsized in order to increase the chance that the PIC approach will be successfully implemented and underscores the importance of assessing implementation outcomes during the preliminary implementation phase [4, 24, 25].

Finally, we found that the GPs’ views on acceptability, appropriateness, and feasibility mainly correlated with agreeing that there is a need for care change, and problems with current care. This implies that, although GPs recognize the need of patients with complex needs, the awareness for care change should first be present before we consider looking further into how this approach should be implemented [29].

### Implications for research and/or practice

During the preliminary implementation phase of the PIC or similar approaches, we first recommend an assessment of the awareness of care professionals regarding their need for care change, as this seems to be a relevant correlation with implementation outcomes.

In order to increase the chance of implementation success of such an approach it is important to have a closer look at the factors that according to GPs hinder feasibility in order to increase the chance of implementation success of such an approach. Specifically, we need to take the heavy workload of professionals and the added financial and time investment of this approach into account. Seeing that the heavy workload was repeatedly mentioned as a factor influencing some GPs’ opinions on this approach, we particularly take this into account throughout the different stages of implementation and aim to reduce the burden for GPs as much as possible.

### Strengths and limitations

A strength of the study is that it addresses important implementation factors and precursors of accepting care change before testing the intervention, which is not always done and may influence adoption of interventions in practice [24–26]. Furthermore, these factors were studied among the GPs -gatekeepers of healthcare- providing us with important insights that will guide us in increasing the implementabillity of this approach.

One limitation is that the current pilot survey had, due to its exploratory nature, a small sample size and narrow focus on two specific regions in the Netherlands, which limits the generalizability of our findings. Although age and gender are comparable with the whole GP group in these regions, more GPs from urban areas were included, which limits the generalizability of the results to more rural areas. Although, the results should be interpretated with caution, this study does provide a first insights in the perceptions of our targeted GPs regarding patients with complex needs and the PIC approach. A second limitation is that most of our constructs were assessed using limited or even single items. This was done in order to limit the demand and make participation more feasible for GPs, considering their already heavy workload. The third limitation is that the open questions unfortunately did not yield the desired richness we expected. Consequently, this limits our ability to provide a more comprehensive analysis combined with the quantitative data. A final limitation is that we asked GPs to self-assess their ability to recognize our target group and their needs instead of asking them in detail about the characteristics of this group or the content of their needs. Although our results show a clear indication of the GPs’ perceptions regarding the PIC approach, we recommend to conduct more in depth analysis to validate our findings.

## Conclusion

This exploratory pilot survey provides an initial insight that GPs recognize patients with complex needs, but that their needs are not yet fully understood. Most GPs indicated a clear preference towards care change for these patients. However, the implementation of an alternative approach like the PIC approach, is something these GPs are still divided on. According to the GPs, even though the implementation of such an approach is needed, it still requires improvements mainly pertaining to its feasibility. Although, exploratory findings should be interpretated with caution, it still offers valuable directions for refining the intervention and inform future large-scale implementation studies.

## Supplementary Information

Additional file 1. Proactive care approach hotspotters questionnaire.

## Data Availability

The datasets generated and/or analysed during the current study are available in the Open Science framework repository, [https://osf.io/8abxj/?view_only=dbe47312bf80442f89331e035851d682].
