# Supplementary material for: The perceptions of Dutch general practitioners on the implementation of a proactive integrated care approach for patients with complex needs: a pilot survey
Source: BMC Prim Care. 2026 Jan 16;27:56. doi: 10.1186/s12875-026-03176-x (PMC12896037; doi:10.1186/s12875-026-03176-x)
Supplement: Supplementary file 1 — Additional file 1. Proactive care approach hotspotters questionnaire. [file 12875_2026_3176_MOESM1_ESM.pdf]

Proactive Care Approach Hotspotters Questionnaire

A. Start of the questionnaire

If you are participating in the hotspotters project, you do not need to complete this questionnaire.

Are you currently participating in the hotspotters project? (Yes/No)

B. Information letter about the study and data handling

Dear GP,

Thank you for your interest in this implementation study at [blinded for peer review].
The aim of this study is to find out whether an alternative care approach for a complex group of patients can be widely implemented in the Netherlands and what is needed to do so. The approach focuses on patients who are known as 'hotspotters': patients who have a complex care question due to multi-problems and make frequent use of (acute) care. The care approach we examine is proactive and integrated in its nature. This implies fostering cooperation between disciplines and looking together with the patient at what is needed for health improvement. To gather information on the implementability of this approach, we ask you to complete an online questionnaire once. You will also receive a short letter with more information about hotspotters and the proactive integrated care approach. We ask you to read the information carefully before completing the questionnaire.

The data collected will be processed anonymously and in compliance with the GDPR law. Furthermore, the data will be stored on a secure drive at [blinded for peer review] for 15 years, in accordance with EU Regulation 2017/745.

The data we collect for this study is intended for a scientific publication. In the article, we will express our thanks to the participants. This will be done completely anonymously. The article for which this data is collected will be shared with you by email after publication.

Reading the information and completing the questionnaire will take +/- 15 minutes and participation in this study is voluntary.

If you have any questions about this study, please contact: [blinded for peer review]

C. Informed consent

By clicking on the box below, you indicate your agreement with the following points:

- I was informed about the study.
- I read and understood the information about the study.
- I had the opportunity to ask questions about the study.
- I have had the opportunity to think about participating and understand that participation is voluntary.
- I understand how the data will be processed (anonymously).
- I am currently working as a GP in the Netherlands.

- *I agree with the above points.*

D. Start of the demographic section

1. Have you previously been approached to participate in the hotspotters project? (Yes/no)

2. In which municipality is the practice you are associated with located? (open ended).

3. Where is the practice you are associated with located? (Urban area/ Rural area).

4. What is your gender? (Female, male, non-binary or I would rather not say)

5. What is your age? (open ended)

E. Information letter about the Proactive Integrated Approach and Hotspotters

Before we ask you some questions about the proactive integrated approach and patient group, we want to give you more information about this care approach. We ask you to read this information carefully before answering the questions.

The proactive and integrated care approach for hotspotters

Hotspotters are patients with complex care needs due to a combination of chronic physical conditions and social or psychological problems. These patients also frequently utilize acute care and the current care offer, which is often reactive, does not seem to adequately meet the needs of this patient group.

To help this target group, we want to introduce a proactive and integrated care approach that looks broadly at the patient's health status. This involves encouraging collaboration between care disciplines and including the patient in the care process to understand what is needed for health improvement. The broader picture of the patients health status is obtained by examining (with the patient) how they are functioning in different life domains.

Currently, this care approach is being implemented in a number of practices in Zoetermeer, Nijmegen and The Hague. Through this study, we want to explore what is needed to implement this proactive and integrated care approach more widely in the Netherlands in the future.

What does this approach entail?

As indicated earlier, hotspotters experience problems in two of the following domains: social, somatic or psychological.

For this reason, it is important that a collaboration between GP, the mental health nurse practitioner (in dutch abbreviated as POH-GGZ) and professional from the social domain is possible for the implementation of the care approach.

Below you can read step by step what the care approach will look like.

1. Intake interview:
   The patient has an intake with the POH-GGZ (45-60min) based on the Positive Health Spiderweb, which focuses on evaluating six dimensions: bodily functions, mental well-being, meaningfulness, quality of life, participation and daily functioning. This gives the patient and professionals involved a detailed picture of the patient's health status.
2. A multidisciplinary meeting (MDM) with the GP, POH-GGZ, social worker and the patient: During this MDM, a personal care plan is constructed, which includes at least two care goals that are formulated by the patient. Furthermore, a care coordinator from one of the three care domains (social, somatic or psychological) is assigned to the patient.
3. Implementation of the care plan:
   The care coordinator maintains contact with the patient and assists the patient in achieving the care plan goals. The frequency and approach will vary from patient to patient. The process with the care coordinator starts immediately after the first MDM.
4. Follow-up MDM(s):
   After the first MDM, at least 1 follow-up MDM takes place to discuss the patient's care situation together with the patient and the whole team. The number of follow-up MDMs will vary per case. If not all professionals can be present during the MDM, it is important that at least 1 professional who is trained in the positive health methodology is present, as this methodology forms the basis in this care approach.

F. Start questionnaire

We request you to continue answering the questions if you have the read through information letter on the care approach.

The questionnaire consists of 23 items.

The following statements are about how acceptable, appropriate and feasible you find the new approach.
You should indicate the extent to which you disagree or agree with each of the statements.

1= completely disagree

2= disagree

3= Neither agree of disagree

4= agree

5= completely agree

1. This new care approach meets my approval.
2. This new approach is appealing to me.
3. I like this new care approach.
4. I welcome this new care approach.
5. This new care approach seems fitting.
6. This new care approach seems suitable.
7. This new care approach seems applicable.
8. This new care approach seems like a good match.
9. This new care approach seems implementable.
10. This new care approach seems possible.
11. This new care approach seems doable.
12. This new care approach seems easy to use.

<u>G. Questions about the organizational capabilities of the general practice and recognition of the patient needs</u>

You can answer the following statements by answering yes or no.

13. In the practice where I work, it is possible to organize MDMs
14. In the practice where I work, there is a POH-GGZ employed
15. In the practice where I work, there is collaboration with professionals from the social domain.
16. In the practice where I work, I recognize the target group (hotspotters).

<u>H. Questions about the recognition of the positive and problem factors of the current care approach.</u>

You can answer the following statements by using the following Likert scale

1= completely disagree

2= disagree

3= Neither agree or disagree

4= agree

5= completely agree

17. I am aware of the needs of this target group.
18. I am aware of the factors that positively influence the care process of this target group.
19. I am aware of the problems present in the care process of this target group.
20. I believe the care approach of this target group needs to change.

I.Open ended questions about acceptability, appropriateness, and feasibility.

In conclusion, we ask you to answer the following open-ended questions.

21. Can you indicate why you find the care approach acceptable or not?
    (Additional explanation: here you indicate whether you find the approach adequate and whether it appeals to you. If you do not find the approach acceptable, you can also indicate what is needed to make it acceptable.
22. Can you indicate why you think the care approach is or is not appropriate?
    (Additional explanation: here you indicate whether you consider the approach appropriate, applicable and suitable for primary care (GP practices) and for health improvement of the target group. If you feel the approach is not suitable, you can also indicate what is needed to make it appropriate.
23. Can you indicate why you consider the care approach feasible or not?
    (Additional explanation: here you indicate whether you think the approach can be implemented in GP practices. Consider whether it is easy to use and feasible. If you think the approach is not feasible, you can also indicate what is needed to make it feasible.

J. Ending of the questionnaire

Thank you for taking the time to participate in this survey.

We conduct research on the feasibility, acceptance and appropriateness of the proactive integrated care approach according to GPs in the Netherlands.

We are also curious to know what the organizational opportunities are within your general practice and how the recognition of hotspotters is among GPs. The questionnaire you have just completed will only be administered once.

If you have any questions about the study or wish to withdraw your consent to participate, please contact [blinded for peer review].

You can also contact us if you wish to participate in the hotspotters project to implement the proactive integrated approach in your general practice.
